# River plastic hotspot detection from space

**DOI:** 10.1016/j.isci.2025.114570

**Published:** 2025-12-29

**Authors:** Ámbar Pérez-García, Graciela Amanda, José F. López, Marc Rußwurm, Tim H.M. van Emmerik

**Affiliations:** 1 Institute for Applied Microelectronics, University of Las Palmas de Gran Canaria, 35001 Las Palmas de Gran Canaria, Spain; 2 Hydrology and Environmental Hydraulics Group, Wageningen University, 6708 BP Wageningen, the Netherlands; 3 Geo-information Science and Remote Sensing Laboratory, Wageningen University, 6708 BP Wageningen, the Netherlands

**Keywords:** Earth sciences, Environmental science, Pollution, Remote sensing

## Abstract

Plastic pollution threatens terrestrial and aquatic ecosystems, and rivers play a central role in transporting and retaining plastics across landscapes. Effective mitigation requires scalable methods to identify riverine plastic accumulation hotspots. Here, we present a semi-automated, cloud-based pipeline that integrates satellite remote sensing and machine learning to detect river plastic hotspots. High-resolution PlanetScope imagery is used to annotate training regions, which are transferred to Sentinel-2 multispectral data to train Random Forest classifiers within Google Earth Engine. The approach is evaluated across three contrasting river systems—the Citarum (Indonesia), Motagua (Guatemala), and Odaw (Ghana)—to assess transferability under diverse environmental conditions. Intra-river transfer achieves up to 99.5% accuracy, while optimized inter-river transfer yields a plastic F1-score of 79%, outperforming previously reported results of 69%. By providing an open-access Google Earth Engine application, this work enables reproducible, large-scale monitoring of riverine plastic pollution and supports the development of global, satellite-based assessment strategies.

## Highlights

• Developed a GEE-based workflow for river plastic detection using Sentinel-2 data
• Used PlanetScope imagery to generate training ROIs for classifier development
• Achieved up to 99.5% intra-river accuracy and 79% F1-score in inter-river transfer
• Released an open-access application for global Sentinel-2 based plastic monitoring

## Graphical abstract

**Figure:**
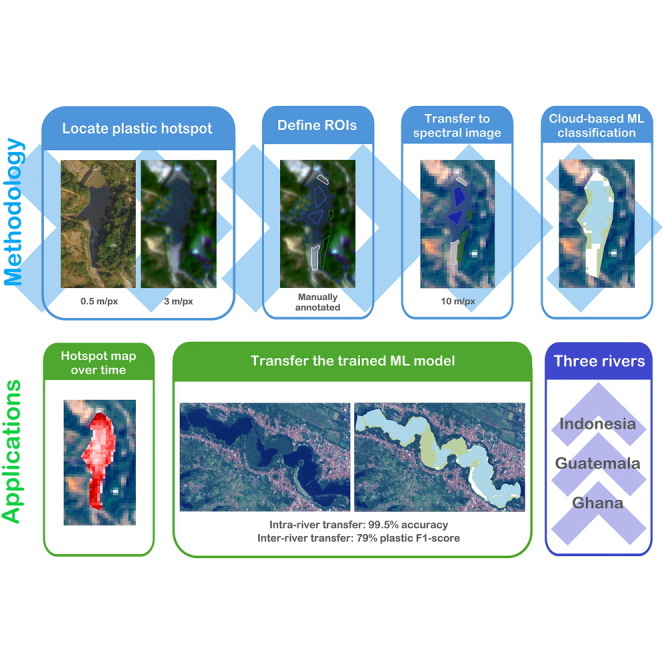

## Introduction

Plastic pollution is a growing global concern that threatens terrestrial and aquatic ecosystems, species, and human health and livelihood.^1^^,^^2^ Through initiatives such as the Global Plastics Treaty, governments are committing to the reduction of environmental plastic pollution.^3^ To effectively reduce plastic pollution, understanding the sources, pathways, and sinks of plastic pollution is key.^4^ Rivers connect the terrestrial and marine environment, and therefore play an essential role in the global distribution of plastics. Plastics can be retained within rivers for extended periods and may be exported to the sea. Plastic transport and retention dynamics strongly depend on the plastic item properties and river characteristics.^5^^,^^6^ Jointly, they determine whether plastics remain mobile and travel long distances, or accumulate on floodplains, within the sediment, or around coastal zones.^7^ Plastic transport and retention dynamics strongly depend on the plastic item properties and river characteristics.^5^^,^^6^ Together, these factors determine whether plastics remain mobile and travel long distances, or accumulate on floodplains, within the sediment, or around coastal zones.^7^ Reliable monitoring of plastic pollution across river compartments is crucial for identifying accumulation hotspots, establishing baseline pollution levels, and evaluating the impact of plastic reduction strategies.^8^^,^^9^ However, *in situ* monitoring of plastic pollution can be labor and cost-intensive and often unsuitable during and after extreme events.

Satellite remote sensing offers a potential avenue to upscale plastic detection efforts^10^ due to the distinct spectral reflectance of plastic compared to water, vegetation, sand, and other materials.^11^ The spectral signatures also vary with polymer type and state of weathering.^12^^,^^13^^,^^14^ Multi- and hyperspectral imagery has been successfully used to detect and classify plastics under laboratory and field conditions, often using (combinations of) spectral indices.^15^^,^^16^^,^^17^^,^^18^ Although the detection can be affected by several physical and environmental constraints such as cloud cover, seasonal variability, and illumination conditions, various satellite remote sensing-based approaches have been developed for the direct and indirect detection of plastics on land, in the ocean, and in rivers.

In recent years, significant progress has been made in the satellite-based detection of plastic in both inland and coastal environments.^19^^,^^20^ Optical and multispectral satellite data, particularly from Sentinel-2, have been widely applied to detect floating or stranded plastics using spectral indices and machine learning models. Several studies have focused on marine and coastal settings,^21^^,^^22^^,^^23^ while others explored inland or riverine scenarios. Among these,^24^ demonstrated the potential of Sentinel-2 time series for detecting floating debris on inland waters, and^25^ applied supervised machine learning with very high resolution (VHR)-assisted labeling to map riverine litter, discussing the 10 m spatial resolution limitation for small debris patches (<100 m^2^).^26^ validated an adjusted Plastic Index for highly polluted rivers, and regional-scale analyses have identified waste hotspots and river blockages using multispectral data.^27^ Hybrid approaches integrating citizen science and remote sensing have also emerged, linking satellite observations with *in situ* river monitoring.^28^

Building on these developments, recent advances in cloud-based processing have enabled the scalable implementation of such methodologies. Platforms such as Google Earth Engine (GEE)^29^ and Microsoft Planetary provide access to extensive multi-temporal satellite archives and powerful cloud computing resources, allowing the large-scale execution of machine learning algorithms without the need for local data storage.^30^^,^^31^^,^^32^ In plastic monitoring, GEE has already been employed to map floating plastics in rivers^19^ and in marine environments.^33^

This work evaluates the potential of a semi-automated pipeline for detecting plastic patches and hotspots in river systems, integrating satellite imagery and machine learning within a reproducible workflow. The core classification and detection process is implemented in Google Earth Engine (GEE), enabling a scalable application using Sentinel-2 multispectral data. High-resolution PlanetScope imagery is used in the initial, external step to manually annotate training regions of interest (ROIs), enhancing spatial precision. Although this annotation step is not automated and requires access to commercial data - limiting its scalability - it serves as a one-time calibration phase. Once ROIs are uploaded to GEE, the classification pipeline can be generalized and applied globally using pre-trained models. Auxiliary field-based or observational data are used in this study to interpret and validate results, but they are not part of the automated pipeline. We apply our methodology to three river systems with varying characteristics: the Citarum River in Indonesia, the Motagua River in Guatemala, and the Odaw River in Ghana. Each river represents different environmental, climatic, plastic hotspot composition, and social contexts. We evaluate both the classification performance and transferability of the models across sites. We also used our methodology for a time series analysis to produce hotspot maps and developed a general model with reduced features that support future field monitoring and intervention planning. We present a companion GEE application that allows end-users to apply the trained general model on new rivers worldwide, directly using Sentinel-2 imagery. This operational tool extends the study’s impact by enabling the scalable application of a validated model, providing a bridge between research and field-ready plastic monitoring.

The key innovation of this study lies in its approach to building and evaluating generalizable river plastic classifiers based on the synergy between spatial detail and spectral richness. By combining expert-led manual annotation from high-resolution imagery with systematic feature analysis and machine learning in a cloud environment, we demonstrate that high-performance plastic detection is possible even with medium-resolution Sentinel-2 data. Previous studies have demonstrated the potential of remote sensing for plastic detection in aquatic systems.^34^ A recent study estimated fractional plastic coverage using high-resolution PlanetScope and Skysat imagery,^19^ while others exploit spectral indices for plastic detection.^35^ These efforts highlight the importance of both spatial detail and spectral analysis in detecting plastic features. Building on this foundation, our work integrates these insights into a scalable, cloud-based workflow tailored specifically for riverine environments. We use high-resolution PlanetScope imagery to annotate training data and leverage Sentinel-2 multispectral data within Google Earth Engine for classification. Our approach extends the methodology to diverse river systems by incorporating spectral indices, band importance analysis, and inter-river transfer tests. This is the first study to combine high spatial and spectral information in GEE, demonstrating its feasibility for developing general plastic detection models while also identifying current limitations and paths forward for model robustness and scalability.

## Results

For clarity, in the following sections, we refer to each study site by the name of its country (Indonesia, Guatemala, and Ghana). However, the results correspond to specific river sections—Citarum, Motagua, and Odaw—where plastic accumulation hotspots have been previously documented and described in detail in STAR Methods section.

### Spectral characteristics of river plastic

**Figure 1:**
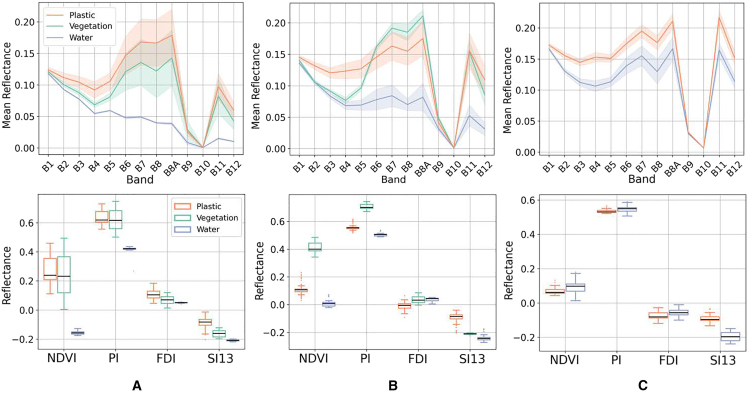
Spectral analysis of datasets In the first row, the mean spectra with their standard deviation, and in the second row, the indices boxplot. (A–C) indicates the area of study in each column. (A) Indonesia, (B) Guatemala and (C) Ghana.

In the first experiment, we analyzed the spectral profiles of the three target classes (plastic, water, and vegetation) across the riverine environments in Indonesia, Guatemala, and Ghana. The first row of Figure 1 presents the mean reflectance spectra, highlighting overall similarity among classes with region-specific variations. In Indonesia, both the Plastic and Vegetation classes exhibit a higher standard deviation, indicating greater spectral heterogeneity. This variability likely arises from mixed pixels containing both plastic debris and surrounding organic material. In Guatemala, spectral curves show lower intra-class variability, and Vegetation displays a pronounced peak in the near-infrared region, suggesting the presence of healthy flora. Ghana yields highly similar spectral responses for Plastic and Water. This convergence is attributed to the narrow geometry of the river, where pixel mixing reduces the ability to distinguish between surface materials.

To improve class separability, several spectral indices have been implemented (second row of Figure 1). Among these, ${\text{SI}}_{13}$ offers the most consistent separation, being the only one that can improve discrimination in Ghana. NDVI and PI perform well in Guatemala, which overall presents the highest separability across all computed indices. NDVI is also the index that best distinguishes water from other classes in both Indonesia and Guatemala. In contrast, the FDI lacks discriminatory capacity, indicating limited applicability for detecting plastic accumulations in fluvial environments. To better assess the spectral separability of the three classes, the NDVI is plotted against ${\text{SI}}_{13}$. These two indices demonstrated the highest discriminative power and can complement each other.

**Figure 2:**
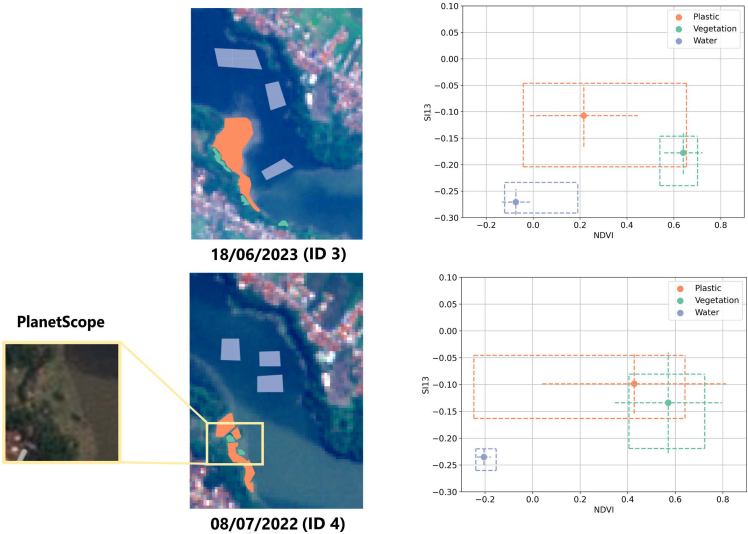
NDVI and ${\text{SI}}_{13}$ values for each class The center dot represents the mean index value for each class, with the dashed lines representing two standard deviations. The dashed outer lines represent the minimum and maximum values of the indices for each group.

To illustrate the spectral variability produced by mixed debris within the plastic accumulation patches, Figure 2 shows images ID 3 and ID 4 from Indonesia, including the polygons manually delineated. Each point represents the mean index value for a class, along with its standard deviation and minimum and maximum bounds to visualize intra-class variability and inter-class overlap. These two scenes were selected to exemplify how the plastic patch composition alters its spectral response, and therefore, class separability. In ID 3, vegetation primarily appears along the riverbanks, and the plastic patch appears homogeneous. As a result, although the maximum values of the vegetation and plastic classes partially overlap, their mean values are distinct, and the standard deviations do not intersect, indicating that these classes can be statistically separated. In contrast, ID 4 reveals green dots within the plastic patch, likely due to embedded vegetation. This leads to greater spectral variability and overlapping standard deviations between the plastic and vegetation classes. This observation raises questions about the purity and composition of the plastic patches—issues that are further analyzed in the discussion section.

### River plastic hotspot detection

**Figure 3:**
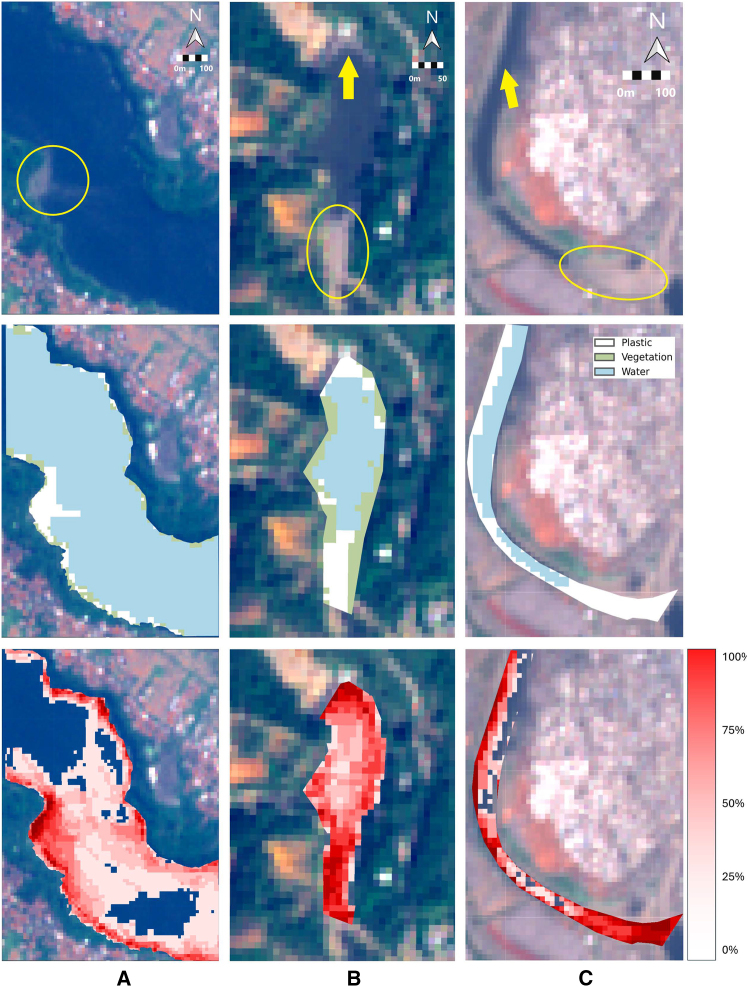
Sentinel-2 imagery of riverine plastic pollution Top row: representative image per river with plastic patches highlighted in yellow and annotated scale (same for each column). Middle row: scene classification maps. Bottom row: hotspot maps from ten-image aggregates per location. (A–C) indicates the area of study in each column. (A) Indonesia, (B) Guatemala and (C) Ghana.

The Random Forest (RF) classifier was trained on image IDs 1–6 and tested on IDs 7–10 for each river, all representing different dates of the same hotspot section. The performance of the model on each river is shown in Figure 3, with the true-color image in the first row and the classification map overlaid on the second row. The three selected images are from the test sets and show how well the classifier performs in identifying the different coverages. The quantitative analysis is presented in Table 1, which displays the overall accuracy or F1-score of the Plastics class for the three rivers. Also in this table are the results not only for the classification using the satellite bands (LA2 B1-12), but also the performance when including the spectral indices (NDVI, PI, FDI, ${\text{SI}}_{13}$). By also providing the indices to the classifier, performance improves in all cases, especially in Indonesia and Ghana, where it increases by more than 20%. This confirms the discriminatory capacity of the indices to detect plastic accumulation mentioned in the previous section.

**Table 1:** River classification metrics

		Dataset		
		Indonesia	Guatemala	Ghana
Only Bands	Overall Accuracy	98.8%	76.7%	70.0%
	Plastics F1-score	71.8%	71.9%	73.7%
Bands + Indices^a^	Overall Accuracy	99.3%	83.6%	73.0%
	Plastics F1-score	98.5%	91.9%	76.6%

a Individual river performance using the bands and also including the spectral indices.

Hotspot maps can be obtained by generating classification maps for various dates, and the areas with the highest frequency of plastic accumulation can be identified. As shown in the river hotspot maps in Figure 3, plastics tend to accumulate in the river bends. In this case, the percentage represents the proportion of images in which plastic was detected at each location, with 100% indicating detection in all 10 images of the dataset for a given river. In Guatemala, plastics accumulate mainly at both ends of the reservoir. In Ghana, accumulation can be observed at the downstream end of the river section, where the river course is affected by a water-control weir. The left side of the river is also highlighted; however, this may be influenced by pixels mixed with adjacent infrastructure.

### Band importance for the classifier

**Figure 4:**
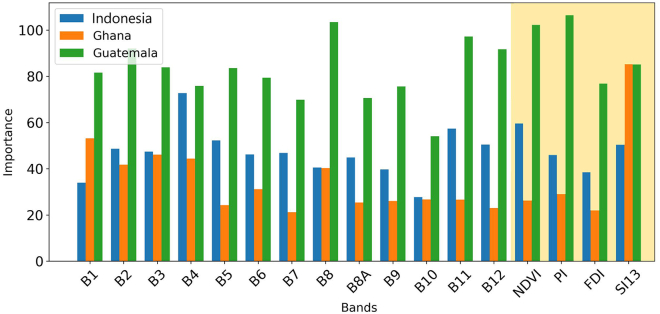
Comparison of the importance of bands and indices for the RF model by river

A key aspect of understanding classifier learning is evaluating the relative influence of the spectral bands and indices used by the model. The feature importance of the classifier shown in Figure 4 represents the absolute contribution of each variable by measuring for each decision node in the RF how much the impurity increased based on the respective variable. RF importance is dimensionless and dataset-dependent; normalizing it will lose the magnitude of importance. The importance rankings reveal clear regional differences in the importance of spectral features. For instance, the most influential band in the Indonesian model is Band 11, whereas Band 1 and Band 2 are the most critical for Ghana and Guatemala, respectively. When extending the analysis to include spectral indices alongside the bands, the feature hierarchy shifts. In particular, ${\text{SI}}_{13}$ is more relevant than any other band for Ghana, suggesting that indices can better capture class-specific spectral responses under certain conditions.

### Generalizability of the river plastic detection method

A key aspect determining the applicability and generability of the presented model is its transferability capacity across diverse environmental settings. Given the inherent variability in riverine systems, it is essential to evaluate how well a model trained in one context performs when applied to another.

### Successful transference to another area within the river

**Figure 5:**
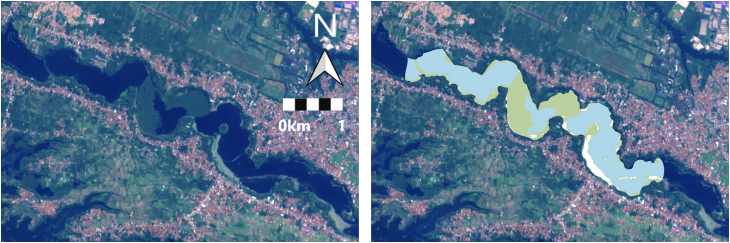
Sentinel-2 image and classification map transferring the model within the same river

The first step in evaluating model transferability involved applying a model trained on one section of the Citarum River in Indonesia to a separate downstream location. For this purpose, six Sentinel-2 images were processed using all the spectral bands, and classification was carried out without any additional tuning. As shown in Figure 5, both visual assessment and quantitative metrics confirm a highly successful transfer, achieving an overall accuracy of 99.5%. On a class-by-class basis, performance remained robust, achieving F1-scores of 99.9% for Water, 99.3% for Vegetation, and 96.1% for Plastic. This underscores the model’s capacity to effectively generalize in comparable fluvial settings, despite a slight rise in misclassification between Plastic and Vegetation due to their shared spectral characteristics. Importantly, this validation was conducted on a much larger dataset than that used for training, with thousands of pixels per class—7767 for Water, 3696 for Vegetation, and 669 for Plastic—compared to the few hundred or fewer pixels typically available in the training zone.

### Improving generalization between rivers with spectral indices

Transferring the model between different rivers presents a greater challenge due to the distinct environmental and spectral characteristics of each region. To systematically evaluate inter-river transferability, models were independently trained using the 10 available images per river dataset and validated on the corresponding 10 images of the target river. The results shown in Table 2 highlight the limitations of model generalization under diverse conditions. Table 2 excludes transfer within the same river, as retraining the models using the ten available images per river maximizes the use of spectral variability for learning. However, this approach leaves no independent data for intra-river validation, making it unsuitable for evaluating without risking overfitting.

**Table 2:** River classification performance metrics for the transferred model. Overall Accuracy and F1-score for the sensor bands, and also including the spectral indices for each dataset.

		Train		
		Indonesia	Ghana	Guatemala
Test (Only Bands)	Indonesia	–	71.4%/61.3%	54.5%/43.5%
	Ghana	54.1%/68.6%	–	89.1%/84.1%^a^
	Guatemala	51.6%/62.1%	49.9%/57.1%	–
Test (Bands + Indices)	Indonesia	–	87.9%/87.1%^a^	85.4%/77.2%^a^
	Ghana	59.3%/68.1%^a^	–	59.2%/71.6%
	Guatemala	75.2%/84.2%^a^	69.9%/85.8%^a^	–

a Overall Accuracy and F1-score for the sensor bands, and also including the spectral indices for each dataset.

Using only the bands to train the models, solely two of the transfer scenarios achieved OAC values exceeding 70%. Furthermore, repeated runs of the same model configuration yielded inconsistent outcomes, demonstrating a high degree of variability and underscoring the lack of robustness in these cross-domain applications. These findings suggest that while intra-river transfer is feasible, inter-river generalization remains limited under current conditions and may require tailored domain adaptation strategies or additional data harmonization to improve stability and accuracy.

To enhance model performance across diverse riverine environments, spectral indices were incorporated into the classifiers as additional input features. This decision was based on the spectral analysis results, which exhibited that specific indices offered improved separability between classes. As shown in the second row of Table 2, the inclusion of spectral indices led to a notable improvement in classification metrics across nearly all transfer scenarios. All model transfers showed enhanced accuracy and F1-scores, except for the Guatemala-to-Ghana case.

In several cases, the gain exceeded 20%, underscoring the utility of combining spectral bands with derived indices. The transfer from Guatemala to Ghana showed an improvement of nearly 30% in the Plastic class F1-score, highlighting the ability of indices to compensate for spectral variability introduced by regional differences. Therefore, by combining the most predictive band or index for each river in the RF classifier, the model achieves an average F1-score of 79% for the plastic class. These results confirm that augmenting the feature space with targeted spectral indices substantially improves the robustness and transferability of plastic detection models across diverse fluvial systems.

### Toward a general model

An alternative approach to improve the generality and robustness of the model involves training a general model using a composite dataset that incorporates data from multiple river environments. To this end, a combined training dataset was constructed using the first two images (ID 1 and 2) from each of the three rivers. Details of this combined dataset can be found in the Workflow implementation section.

Several experiments have been conducted to evaluate the performance of the general model under various conditions. The results shown in Table 3 confirm that incorporating spectral indices generally enhances model performance. However, an even greater improvement is achieved by selecting a minimal set of highly informative spectral features, obtained from the Band importance for the classifier section. This targeted feature selection reduces the introduction of noise and redundancy from less relevant features, thereby improving classifier precision and stability. Among the evaluated configurations, the selection of the most important spectral bands (B1, B2, B11) yielded the highest classification accuracy for individual rivers. Nonetheless, this setup also produced the lowest F1-score for the Plastic class in the second Indonesian location, indicating a trade-off between peak accuracy and consistency across environments. The configuration based on the most relevant features—combining bands and indices (B1, B2, ${\text{SI}}_{13}$) provides the best overall performance across datasets, offering a strong balance between generalization and precision.

**Table 3:** General model classification metrics

		Dataset				
		Indonesia	Ghana	Guatemala	Indonesia 2	Average
Only Bands	OAC	94.2%	55.2%	86.9%	94.9%	82.8%
	F1	97.8%	60.6%	84.4%	89.8%	83.2%
Bands + Indices	OAC	95.8%	62.1%	89.3%^a^	97.3%	86.1%
	F1	97.5%	64.5%	87.7%	95.8%^a^	86.4%
Bands B1 B2 B11	OAC	96.9%^a^	100%^a^	89.3%^a^	93.1%	94.8%
	F1	98.1%^a^	100%^a^	92.2%^a^	58.6%	87.2%
Bands B1 B2 Index SI13	OAC	96.6%	100%^a^	88.1%	98.7%^a^	95.9%^a^
	F1	97.9%	100%^a^	91.8%	93.9%	95.9%^a^

a Overall Accuracy and F1-score per dataset for different combinations of bands and spectral indices.

## Discussion

### River plastic hotspot detection with good performance

This study demonstrates that accurate remote sensing of riverine plastic accumulation is feasible using medium-resolution Sentinel-2 imagery, combined with strategic feature engineering and cloud-based classification. By integrating spatial annotations from PlanetScope and spectral indices tailored for plastic detection, we advance a transferable, reproducible, and operationally viable approach to environmental monitoring within Google Earth Engine.

High classification accuracy was achieved in within-river applications, as shown by the 99.5% overall accuracy in the intra-river transfer test on the Citarum River in Indonesia. The model performs exceptionally well in the intra-river transfer scenario, even when evaluated on a significantly larger number of pixels. This strong performance is likely due to the proximity of the two locations on the Citarum River, which share similar environmental conditions, including vegetation types and pollution characteristics, thereby facilitating effective model generalization. Additionally, the use of a hand-curated training dataset likely contributed to this result by minimizing outliers and ensuring high-quality, representative samples for each class.

Inter-river transfers, while more challenging, were significantly improved through the inclusion of spectral indices, with several scenarios showing gains of over 20% in classification performance. These improvements are closely linked to the spectral characteristics observed in each river, where plastic, water, and vegetation display distinct reflectance patterns influenced by environmental conditions such as turbidity, surrounding vegetation, and debris composition. Among the indices tested, ${\text{SI}}_{13}$—specifically designed for plastic detection—consistently ranked as one of the most important features across all locations, enhancing class separability. Additionally, NDVI and PI contributed to classification accuracy in the Guatemalan dataset, where vegetation in the patch introduced variability that these indices helped distinguish.

Prior studies have achieved high accuracy in coastal and marine contexts using multispectral data and machine learning.^19^^,^^36^ However, only a few have addressed the generalizability of these methods across geographically diverse sites. Our workflow builds on previous Sentinel-2 approaches but differs in scope and transferability.^23^ achieved 80–90% accuracy using an RF general model for coastal waters, but requiring *in situ* validation data and without the hydrological complexities of river systems.^24^ employed spectral indices and temporal series analysis to monitor floating debris dynamics and estimate the plastic cover at the subpixel level within a single river system. On the Tisza,^25^ trained several ML models using VHR-assisted labels, with RF, artificial neural network (ANN), and Support Vector Classifier (SVC) achieving the best performance. While validation F1 was high (SVC 0.94, ANN 0.93, RF 0.91), generalization on larger unseen data dropped to medium-poor (RF 0.69, SVC/ANN 0.62) due to single-river dependence. Our inter-river plastic F1 = 79% is therefore competitive under unseen data, while our intra-river tests preserve very high accuracy.

Our work demonstrates that, even with Sentinel-2’s moderate resolution, when integrated with PlanetScope-based ROI annotation, meaningful detection of plastic accumulations is possible, especially when combined with targeted feature selection and cloud-based processing. To support scalability and broader impact, we provide a GEE-based application called Plastic River Classifier (https://plastic-monitoring.projects.earthengine.app/view/river-plastic-monitoring) that allows users to apply the trained classifier to rivers globally. This app serves as a first step toward operational use of our classifier and supporting wider plastic monitoring using generalized models.

### Are we really detecting plastic?

Determining whether the detected targets are truly plastic remains a complex but critical question. In practice, what we identify as “plastic patches” are often aggregations of heterogeneous debris, including various types of plastics, floating vegetation, large wood, and other anthropogenic materials.^37^^,^^38^ These mixtures can vary significantly between rivers and even across seasons within the same location.^7^^,^^39^ This result highlights the importance of awareness of mixed values and how this may impact the training process and classification outcomes. It is important to emphasize that the quantitative estimation of plastic concentration is beyond the scope of this study; we deliberately focused on well-documented polluted rivers where multiple independent studies have confirmed extensive macroplastic accumulation.^19^^,^^40^^,^^41^^,^^42^

Visual inspections and spectral variability in Indonesia indicate accumulations composed of mixed waste, where plastics are mixed with water hyacinths, organic material, and other types of litter. This composition results in increased heterogeneity in the spectral signature, yet characteristic plastic responses remain discernible. In Guatemala, the challenge lies in the dense vegetation, where shadows cast by overhanging canopy elements alter the reflectance profiles of surrounding pixels. The narrow geometry of the Odaw River in Ghana often results in mixed pixels that encompass both the water channel and adjacent land, thereby diluting class separability. The coarse spatial resolution of Sentinel-2 (10 × 10 m) exacerbates the problem of non-pure pixels, as plastic patches share spectral space with surrounding materials and water. Despite these limitations, visual inspection of the images and spectral patterns supports the interpretation that many of the targets identified are mostly plastic. Spectral indices such as ${\text{SI}}_{13}$ have further proven effective in enhancing class separation.

Beyond validating whether the detected targets are truly plastic, our approach also opens new possibilities for understanding how debris accumulates and redistributes within river systems over time. Our methodology has proven capable of differentiating between anthropogenic and organic materials in rivers. By applying this workflow across multiple rivers and seasons, future analyses could reveal how hydrological dynamics, vegetation growth, and flow variations influence the location and persistence of floating debris hotspots. Such insights would contribute to a more comprehensive understanding of river plastic dynamics, enabling better planning of cleanup operations, improved the prediction of accumulation zones, and more efficient allocation of mitigation resources.

### Generalization and scalability

Model generalization across diverse river systems is a critical step toward the global applicability of remote sensing-based plastic monitoring. While intra-river generalization yielded strong results, inter-river model transfer exposed limitations due to variations in plastic patch composition, ecohydrological conditions, and technical limitations, such as the sensor’s spatial resolution. The current selection of rivers includes a wide range of features. In Indonesia, high turbidity and the co-occurrence of plastics with floating vegetation create mixed spectral signals that increase classification uncertainty.^20^ In Guatemala, clearer water improves class separability, although canopy shadows from riparian vegetation locally reduce accuracy.^19^ In Ghana, narrow channels and nearby built-up areas cause pixel mixing, limiting detection precision.^43^ We encourage follow-up studies in other river systems to also explore and implement the effect of other factors. Nevertheless, integrating spectral indices opens a new avenue for performance enhancement tailored to class separability. Overall, the generalization performance can be considered positive, especially given the diversity and complexity of the test sites. In particular, the lowest results are consistently observed when the models are tested on the Ghanaian dataset, which is expected given its mixed pixels with surrounding land features. These edge effects complicate accurate classification, highlighting the need for additional strategies—such as unmixing or spatial filtering—to further enhance model generalization in such challenging settings.

The general models trained combining images from several rivers improved performance, suggesting that greater training data diversity enhances generalization capabilities. This finding aligns with the literature advocating heterogeneous datasets for remote sensing model development.^16^ Integrating spectral indices into the classifier, along with strategic band reduction, demonstrably enhances classification performance while minimizing the volume of acquired information. Scaling this approach globally would require the dataset to include more rivers under different conditions and to incorporate ground-reference data for validation.

Another direction for improving the scalability of our approach is to simplify the manual annotation step required for model training. Although unsupervised and self-supervised learning methods are promising, their accuracy currently remains below that of supervised approaches. Nonetheless, recent research is moving in this direction^22^: introduced a Naive Bayes classifier that performs well with limited training samples, and^44^ proposed the SAMSelect algorithm to interpret floating marine debris from Sentinel-2 using a small annotated dataset. Such developments could eventually reduce the dependency on high-resolution commercial imagery and enable semi-automated generation of training data for large-scale.

### Limitations of the study

Despite the promising results of our approach, several technological limitations currently hinder its broader operational deployment. One significant challenge is harmonizing data across multiple satellite platforms. Aligning imagery from Sentinel-2 and high-resolution sources, such as PlanetScope, for the same date and location proved particularly challenging, yet it is essential for accurately delineating training and validation ROIs. In highly dynamic rivers, plastic patches can shift position completely within a few hours due to flow velocity or wind, which may introduce spatial discrepancies between acquisitions. To minimize this effect, all images were carefully reviewed, and only those showing consistent patch alignment were used for annotation and training. Furthermore, manual ROI annotation ensures high-quality training data, but it is time-consuming and requires visual expertise, limiting the scalability of model development across new regions. In the future, semi-automated or unsupervised pre-screening methods could help mitigate these limitations and further streamline the annotation process.

Additional issues include cloud cover and atmospheric interference, which remain persistent obstacles in optical remote sensing. Semi-transparent clouds and cast shadows can introduce spectral noise, which affects both classification accuracy and confidence in predictions. Vegetation and canopy cover can also obscure the river surface, particularly in tropical or forested regions, while seasonal differences such as snow, ice, or extreme illumination angles modify reflectance and complicate the interpretation of spectral signals. During the night, optical systems are inherently limited, restricting monitoring to daylight conditions. Sentinel-2’s 10-meter resolution, while sufficient for identifying larger accumulations, struggles to detect smaller or dispersed patches, especially in narrow or vegetated channels. Furthermore, near-real-time applications are constrained by the temporal resolution and latency of Sentinel-2 data. Sentinel-2 revisits the same location approximately every five to ten days at the equator (with shorter intervals at higher latitudes), and Level-2A images are typically released 24–48 h after acquisition. Therefore, for short-term pollution events, this satellite may not provide the necessary temporal frequency.

To overcome some of these limitations, a multi-sensor approach offers substantial potential. Combining optical data from satellites such as Sentinel-2 and PlanetScope with radar imagery from platforms such as Sentinel-1. Radar systems, which are unaffected by cloud cover or light conditions and are capable of detecting surface texture and moisture changes, can complement optical observations. This can provide significant improvements in temporal coverage and in persistently cloudy regions such as Southeast Asia. Furthermore, emerging sensors, such as ESA’s Copernicus hyperspectral imaging mission (CHIME), promise to deliver high-resolution hyperspectral data, enabling more detailed spectral discrimination of plastics. Additionally, the increasing availability of CubeSat constellations, such as those operated by Satellogic and BlackSky, could provide supplementary high-frequency, high-resolution observations.

### Future directions

This study establishes a semi-automated, cloud-based workflow for riverine macroplastic detection using multispectral satellite data. research should build on this foundation by advancing both fundamental, computational, and practical aspects. A key aspect arising in this study is that band selection enhances classification performance. However, these techniques are not available in the native language of GEE. Developing feature selection techniques compatible with GEE should be explored, as well as exploring the use of other computational environments for our methodology.

Similar to previous studies, we found that many pixels contain mixed materials, resulting in mixed spectral signatures. Spectral unmixing methodologies may be explored in the future to better detect the presence of plastics in such situations. Spectral databases of plastics and other materials are key for this. The current databases^12^^,^^13^^,^^17^ cover only limited plastic polymers, item types, and states of degradation and weathering, and should be expanded for global river environments. Unmixing techniques are computationally intensive and currently limited in GEE. Future improvements in cloud computing may enable its practical application for refining plastic detection by accounting for sub-pixel heterogeneity.

In this study, we limited the detection to whether plastics were present in pixels or not. Future fundamental work may also be used to identify the minimum detectable item size and plastic concentration of current and future satellite sensors. Those insights can be used to develop methods to move from detection to quantification. Field-target experiments will be an important step toward understanding the effects of type size and concentration on detectability. In the marine domain,^45^ have already managed to test different floating litter targets and demonstrate the detectability using Sentinel-2.^24^ estimate plastic cover at the subpixel level within a single river system, employing spectral indices and temporal series. For rivers,^15^ reported results from a pilot target experiment using polyester (PES) sheets and PET bottle targets. Here, the item density of 8 items/m^2^ was not sufficient for detection using Sentinel-2. The sheets of 1 × 30 $m^{2}$ were detected, however. Future work should extend such experiments by testing (i) commonly found items in rivers globally, (ii) a wide range of item densities, including mixtures with other materials, and (iii) both riverbank and river surface backgrounds.

Another avenue toward more accurate quantification using satellites is the parallel collection of ground truth data. We recommend designing calibration experiments in rivers with varying characteristics and collecting *in situ* data on plastic concentration and composition at the river surface and on riverbanks. Additional spectral measurements can be taken using handheld spectrometers or multispectral cameras with the same bands as Sentinel-2 (or other satellites). We specifically encourage focusing on areas with active collection of plastics from rivers, either through volunteer efforts along riverbanks or direct collection from the river surface. Future developments should also integrate collaboration with citizen science initiatives and local stakeholders to identify plastic hotspots. Partnerships with programs such as Plastic Pirates^46^ and Clean Rivers^47^ can provide complementary *in situ* validation data at regular intervals, thereby improving temporal coverage and calibration accuracy. In addition, initiatives such as the plastic cup along the Tisza River^28^ maintain consistent, georeferenced records of polluted areas that could serve as valuable ground truth for model validation and refinement, improving long-term river plastic monitoring.

Finally, we encourage the use of our methodology for long-term and large-scale time series analysis to better understand the factors that drive river plastic and retention. Fundamental studies to date mostly rely on limited measurements in space and time. Our remote sensing-based approach may extend data to multiple years and cover entire river courses. Combining remote sensing-based plastic detection with data on hydrology, river characteristics, and anthropogenic factors may shed new light on the driving mechanisms of plastic entry, transport, and retention in rivers. Such efforts will also improve the scalability and generalization of our plastic hotspot detection method to other river environments globally. Furthermore, the proposed methodology could be extended to various terrestrial and coastal applications, including monitoring anthropogenic debris on shorelines, land-based dumps, and landfill areas, but also for other classification tasks such as vegetation mapping, land-cover change detection, and water-quality or sediment classification in aquatic environments.

### Conclusion

This study presents a scalable, cloud-based workflow for detecting macroplastic hotspots in rivers using freely available Sentinel-2 imagery. By combining high-resolution PlanetScope annotation with spectral feature engineering and Random Forest classification within Google Earth Engine, we demonstrate the feasibility of transferring trained models across distinct river systems. Our results demonstrate high intra-river accuracy (up to 99.5%) and promising inter-river transferability (F1-score of 79%) when utilizing targeted spectral indices, such as SI13. These results are competitive and surpass those reported in similar state-of-the-art studies, such as^25^, who achieved an F1 score of 94% for intra-river and 69% for inter-river generalization. These findings support the development of generalizable classifiers that are robust to environmental variability and pixel mixing challenges, especially in complex fluvial settings. The workflow’s integration into an open-access GEE application ensures operational utility and reproducibility, offering researchers a practical tool for monitoring plastic pollution. Future work should focus on expanding annotated training datasets, improving spectral unmixing techniques, and enhancing spatial resolution to enable finer-scale detection and quantification. Our approach contributes to the growing field of remote sensing for environmental monitoring by operationalizing plastic detection at the river scale, bridging the gap between research and field-deployable solutions.

## Resource availability

### Lead contact

Requests for further information and resources should be directed to and will be fulfilled by the lead contact, Ámbar Pérez-García (ambar.perezgarcia@wur.nl).

### Materials availability

The study generated the Plastic River Classifier GEE application: https://plastic-monitoring.projects.earthengine.app/view/river-plastic-monitoring.

### Data and code availability

• All data reported in this article are available at Mendeley Data: https://data.mendeley.com/preview/tczwvtzmys?a=476f97c0-e367-4d52-8fc7-edc79bb4f4b9 (Database: https://doi.org/10.17632/tczwvtzmys.1).
• The source code (GEE) is available from https://github.com/AmbarJade/plastics-hotspots-identification.git.
• Any additional information required to reanalyze the data reported in this article is available from the lead contact upon request.

## Acknowledgments

This work was funded by the OASIS-HARMONIE project, under contract PID2023-148285OB-C43 from “Proyectos de Generación de Conocimiento” 2023 and the AP-BYTE_2CAP project, under contract 2/MAC/3/6.1/0209_CAP, from Interreg Program (MAC 2021-2027).

## Author contributions

Conceptualization, A.P.G. and T.H.M.E.; methodology, A.P.G.; investigation, A.P.G. and G.A.; writing – original draft, A.P.G. and G.A.; writing – review and editing, M.R. and T.H.M.E.; funding acquisition, J.F.L.; resources, J.F.L. and T.H.M.E.; supervision, J.F.L., M.R., and T.H.M.E.

## Declaration of interests

The authors declare no competing interests.

## STAR★Methods

### Key resources table

**Table:** 

REAGENT or RESOURCE	SOURCE	IDENTIFIER
**Deposited data**		

Polygons from Sentinel-2 images	This paper; Mendeley Data	https://doi.org/10.17632/tczwvtzmys.1

**Software and algorithms**		

Plastic River Classifier – Google Earth Engine application for global use	This paper	https://plastic-monitoring.projects.earthengine.app/view/river-plastic-monitoring
Google Earth Engine (GEE)	Google/Earth Engine Team	https://earthengine.google.com/
Python, version 3.8.10 (via Jupyter Notebook)	Python Software Foundation	https://www.python.org
Source code for data processing	This paper; GitHub	https://github.com/AmbarJade/plastics-hotspots-identification.git

### Method details

#### Overall methodology

This study developed a semi-automated, cloud-based workflow to detect macroplastic hotspots in river systems using multispectral satellite imagery and machine learning. The method integrates high-resolution commercial imagery for manual annotation and medium-resolution Sentinel-2 data for classification within the Google Earth Engine (GEE) environment and using Python, version 3.8.10 (via Jupyter Notebook).

The design of our methodology builds upon earlier efforts that applied Sentinel-2 data and ML models for detecting floating or stranded plastics. In particular,^23^ developed an automated marine floating plastic detection system using Sentinel-2 data, ML models, and indices such as FDI and PI, achieving accuracies of 80–90% in the coastal waters of the Mediterranean. However, in-situ validation data were required to train the models, and unlike marine environments, river systems pose additional challenges due to diverse vegetation types, variable water composition, and turbidity conditions.^24^ demonstrated the utility of Sentinel-2 time-series composites for tracking inland debris persistence using GEE and spectral indices within a single river system.^25^ used VHR images to identify litter spots and tested the generalization of ML classifiers on unseen data under varying hydrological conditions, achieving an F1-score of 69% with RF. These studies inspired our approach but also revealed two key gaps: the lack of better cross-river generalization and the limited reproducibility of local workflows.

**Figure 6:**
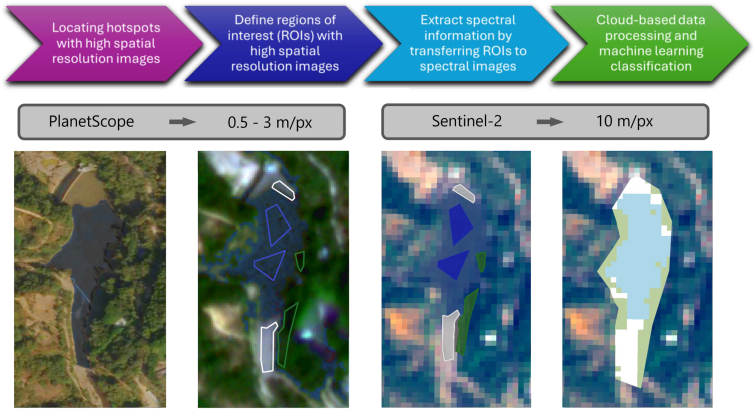
Plastic hotspot detection methodology (1) locating the hotspot, (2) delineating the ROIs in high spatial resolution images, (3) exporting them to the cloud to apply them to high spectral resolution images, and (4) training a machine learning classifier in the cloud. This figure does not have a scale because it is for illustrative purposes

To address these issues, we implemented a cloud-based pipeline in Google Earth Engine that couples high-resolution manual annotation with Sentinel-2 spectral features and optimized band selection, enabling a scalable and generalizable plastic-detection framework. The approach includes the following steps: (1) locating the hotspot, (2) delineating the ROIs in high spatial resolution images, (3) exporting them to the cloud to apply them to high spectral resolution images, and (4) training a machine learning classifier in the cloud (Figure 6). In this study, a plastic hotspot is defined as an area where the surface concentration of visible plastic debris is notably higher than in its surroundings, reflecting localized accumulation driven by hydrological, geomorphological, or anthropogenic factors.^9^ The workflow uses satellite remote sensing, integrating high-resolution imagery, spectral analysis, and cloud-based machine learning. The ultimate goal is to facilitate large-scale and scalable monitoring of plastic pollution across diverse river systems globally.

The first step involves locating macroplastic accumulations, or hotspots, in rivers using high spatial resolution images. This process is entirely manual, relying on local knowledge, satellite imagery, and supporting information from news reports and community observations to identify plastic patches. The spot can be corroborated via Google Earth Pro, which provides a user-friendly interface for visually screening satellite imagery across different periods. In the second step, we manually define regions of interest (ROIs) within these hotspots using VHR imagery, such as SkySat (0.5 m/px) or Dove (3 m/px) from PlanetScope.^48^ These serve as spatial ground-truth references for subsequent spectral analysis and the area where the classifier will be applied is also outlined. The third step entails extracting spectral information by uploading the spatially annotated ROIs to cloud systems. The Sentinel-2 images will be temporally aligned with the high-resolution PlanetScope images in which polygons were delineated to avoid misclassifications. This alignment was critical to minimize misclassifications, although challenges such as water-driven displacement of plastic between acquisitions were noted and factored into data interpretation. This allows for the precise extraction of high spectral resolution data corresponding to accurately located macroplastic patches. Finally, in the fourth step, we conduct a spectral analysis and machine learning classification in the cloud system. Using the labelled pixels, we train a classifier using all the spectral bands and some spectral indices. Model training and testing are fully executed within GEE, leveraging cloud infrastructure to process large volumes of data efficiently and without the need for local storage. This methodology is tested across three different rivers—located in Indonesia, Guatemala, and Ghana—representing varied geographical and environmental conditions. For each river, an individual classifier is trained and evaluated, followed by inter-location and cross-river transfer experiments. This approach enables the construction and evaluation of a generalizable model that can identify plastic accumulations under various scenarios.

#### Study areas and datasets

This study focuses on three rivers located in distinct geographic and environmental contexts: the Citarum River in Indonesia (6.92°S,107.48°E), the Motagua River in Guatemala (14.76°N,90.50°W), and the Odaw River in Ghana (5.54°N,0.22°W). These rivers were selected due to their known high levels of macroplastic pollution and their contrasting environmental characteristics.^40^ Several studies have confirmed these systems as significant plastic accumulation hotspots.^19^^,^^41^^,^^42^ The Odaw flows through the densely populated national capital of Accra, the Citarum faces both high levels of plastics and floating water hyacinths, and the Motagua is impacted by a dam. Together, they represent a diverse set to evaluate model performance and transferability under varying landscape and spectral conditions. Table S1. [Sentinel-2 dataset with the number of pixels identified per class.] contains the information of the Sentinel-2 dataset used to train and test the model. Table S1. [Sentinel-2 dataset with the number of pixels identified per class.] also includes the number of pixels per class obtained with the polygons delineated using the high-resolution imagery, as these are relevant for subsequent sections.

Each study site presents unique environmental and remote sensing challenges. The Citarum River (Indonesia) flows through densely populated urban areas, often accumulating floating waste mixed with organic debris and sediments. This river often has turbid water, and occasionally small sand islands form that can support some vegetation. This mixture results in complex spectral signals, especially for the Plastic and Vegetation classes. In contrast, the Motagua River (Guatemala) traverses a more forested landscape, where dense canopy cover introduces frequent shadows that influence pixel reflectance. The Odaw River (Ghana) presents one of the most challenging conditions due to its narrow channel width, which often leads to pixel mixing with adjacent urban surfaces.

Sentinel-2 imagery, provided openly by the European Space Agency (ESA), offers global coverage with revisit times of 5 days at the equator and has a spatial resolution of 10 m.^49^ The atmospherically corrected Level-2A Sentinel-2 products, generated using the Sen2Cor processor, provide surface reflectance values suitable for land-based applications. While the spectral information is extracted from Sentinel-2 images, spatial information relies on PlanetScope and SkySat imagery. These are commercial products with near-daily revisit capability, offering very high spatial resolution (0.5–5 m).^48^ However, access to PlanetScope and SkySat data requires a subscription or license, which can limit scalability. Through a Campus License we had access to the Planet image archives. During the annotation process, both the RGB and Near-Infrared (NIR) bands from PlanetScope were examined to verify ROI composition.

Despite efforts to temporally align Planet and Sentinel imagery within a two-day window, several challenges emerged across all datasets. One major issue was the displacement of plastic patches caused by water flow or wind, resulting in visible differences between images taken just a few hours apart. Additionally, changes in solar illumination angles introduce shadow patterns, especially under vegetated riverbanks, such as in Guatemala, which complicates class identification. Cloud cover, particularly prevalent in the Indonesian dataset, further introduced noise and data gaps, with semi-transparent clouds or haze affecting spectral accuracy. These factors underscore the difficulty of synchronizing spatial and spectral data across platforms.

#### Workflow implementation

The workflow consists of four main stages: (1) identification of visible plastic accumulation hotspots through manual inspection of high-resolution imagery and supporting field information; (2) delineation of ROIs in PlanetScope imagery; (3) temporal alignment of ROIs with Sentinel-2 scenes within a few day window (depending on the river dynamics); and (4) supervised classification using Random Forest (RF) models in GEE. The training polygons were manually defined and uploaded to GEE. The workflow was implemented entirely in the cloud using the native GEE classifier ee.Classifier.smileRandomForest with 200 decision trees.

To identify large plastic patches for training the algorithm, we focused on rivers known for high plastic pollution levels, leveraging common accumulation zones such as bends, urban discharge points, and informal dumping sites. Polygons representing the ROIs are manually annotated using high-resolution PlanetScope imagery within QGIS and then uploaded to Google Earth Engine, where they are matched with Sentinel-2 images. A total of ten Sentinel-2 images per river were selected, each temporally aligned with the PlanetScope reference imagery. The ten images for each river correspond to different dates at the same location, where hotspots have been confirmed. This pairing strategy enables the extraction of high-spectral-resolution data from spatially accurate regions, thereby enabling precise training of the classification algorithm. The widely used Random Forest (RF)^50^ classifier employs ensemble learning to combine predictions from multiple decision trees.^51^

This multi-layered workflow, built on the foundation of cloud-based processing and optimised spectral input, aims to reduce computational load while increasing the accuracy and reliability of macroplastic detection. To support broader application and scalability, we developed an interactive GEE application that enables users to apply the trained model to Sentinel-2 imagery from any location.

#### Spectral features and indices

Spectral indices are combinations of bands that aim to highlight a property or characteristic of a surface by leveraging the contrasting spectral reflectance between one property or characteristic and another. We used the Normalized Difference Vegetation Index (NDVI), the Sentinel Index 1-3 $\left({\text{SI}}_{13}\right)$), the Plastic Index (PI), and Floating Debris Index (FDI) in our work. Below these indices are briefly explained.

#### Normalized Difference Vegetation Index (NDVI)

The Normalized Difference Vegetation Index (NDVI) combines bands in the red and NIR range to highlight vegetation health:

(Equation 1) $$NDVI=\frac{R_{NIR}-R_{RED}}{R_{NIR}+R_{RED}}$$

The study by Carlson and Ripley^52^ shows that NDVI values are indeed correlated with fractional vegetation cover, such that NDVI is an appropriate method for estimating floating vegetation cover in water bodies. In addition, the study highlighted that NDVI is sensitive to detecting changes in fractional vegetation cover when the vegetation cover is less than 100% in the region of interest. Since full vegetation coverage in river bodies is not a common occurance, applying NDVI in this study to identify floating vegetation is highly opportune.

#### Sentinel Index 1-3 $\left({\text{SI}}_{13}\right)$

Recently, the ${\text{SI}}_{13}$, spectral index combining bands one and three of Sentinel-2 in a normalized difference, demonstrated to be effective for plastic detection from space.^15^

(Equation 2) $$SI_{13}=\frac{R_{GREEN}-R_{AEROSOL}}{R_{GREEN}+R_{AEROSOL}}$$

#### Plastic Index (PI)

The Plastic Index (PI)^18^ was introduced aiming to distinguish plastic litter in the water bodies by leveraging the high reflectance of plastic in the NIR wavelengths:

(Equation 3) $$PI=\frac{R_{NIR}}{R_{NIR}+R_{RED}}$$

The study by Themistocleous et al. (2020) validated the use of plastic float identification using spectral signatures and UAV aerial images, confirming that PI was the optimal index for identifying floating plastic in water bodies. Here, $\lambda_{\text{NIR}}$ represents the near-infrared wavelength (e.g., 859nm) and $\lambda_{\text{RED}}$ represents the red wavelength (e.g., 645nm).

#### Floating Debris Index (FDI)

The Floating Debris Index (FDI)^53^ enhances the detection of floating debris patches in Sentinel-2 imagery:

(Equation 4) $$\begin{array}{ccc} FDI & = & R_{NIR}-R_{NIR}^{\prime }\\ R_{NIR}^{\prime } & = & R_{RE2}+\left(R_{SWIR1}-R_{RE2}\right)\cdot \frac{\left(\lambda_{NIR}-\lambda_{RED}\right)}{\left(\lambda_{SWIR1}-\lambda_{RED}\right)}\cdot 10 \end{array}$$

The FDI quantifies the difference between the observed NIR reflectance and a baseline reflectance derived by linear interpolation between the adjacent RE2 and SWIR1 bands. This approach minimizes atmospheric and observational obstacles, such as glint, aerosols, and solar angle. As a result, it enables the detection of floating materials, including plastic debris, even in the presence of thin clouds or haze.

#### Training and validation strategy

For each river, ten Sentinel-2 images were selected and split into training (IDs 1–6) and test sets (IDs 7–10). The models were initially trained using the 12 bands from L2A Sentinel-2. The total number of training pixels per class is 702, 79, and 659 in Indonesia, 251, 130, and 332 in Guatemala, and 201, 0, and 302 in Ghana, respectively, for the water, vegetation, and plastic classes. For the test class, we have 1095, 167, and 788 in Indonesia, 240, 159, and 261 in Guatemala, and 134, 0, and 124 in Ghana.

To evaluate generalization capacity, the model is trained using two images (IDs 1 and 2) from each river and validated on a test set comprising three images: one from each river (ID 7) and an additional location for the Citarum River in Indonesia (ID 11) with a much larger classification area. Cross-river transferability was evaluated by training on one river and testing on another. Intra-river validation was performed on an independent section of the Citarum River (ID 11), which is larger for testing and has 7,767 pixels for water, 3,696 for vegetation, and 669 for plastic.

#### Feature selection

To improve classification performance, the spectral indices listed previously—known to enhance class separability—were also incorporated as input features. The relative importance of each spectral band and index was computed using the RF feature importance metric in GEE. The most relevant features (B1, B2, and ${\text{SI}}_{13}$)) were used to construct a reduced general model that achieved improved balance between precision and robustness across rivers due to the reduction of redundancy and spectral noise.

### Quantification and statistical analysis

Model performance was quantified using Overall Accuracy (OAC) and F1-score metrics, calculated from confusion matrices. No randomization, blinding, or inferential statistics were required as the study relied on satellite data and deterministic classification. All statistical parameters, including per-class F1-scores, are reported in the results section and Tables 1, 2, and 3.

### Additional resources

The trained classifier and public application are available at: **Plastic River Classifier:** https://plastic-monitoring.projects.earthengine.app/view/river-plastic-monitoring.

Further details and open-source code is available through the corresponding author.

## References

[1] MacLeod M., Arp H.P.H., Tekman M.B., Jahnke A. (2021). The global threat from plastic pollution. Science.

[2] Villarrubia-Gómez P., Carney Almroth B., Eriksen M., Ryberg M., Cornell S.E. (2024). Plastics pollution exacerbates the impacts of all planetary boundaries. One Earth.

[3] Arora H., March A., Nieminen L., Shejuti S.M., Walker T.R. (2024). Plastics.

[4] Morales-Caselles C., Viejo J., Martí E., González-Fernández D., Pragnell-Raasch H., González-Gordillo J.I., Montero E., Arroyo G.M., Hanke G., Salvo V.S. (2021). An inshore–offshore sorting system revealed from global classification of ocean litter. Nat. Sustain..

[5] Mellink Y.A.M., van Emmerik T.H.M., Mani T. (2024). Wind-and rain-driven macroplastic mobilization and transport on land. Sci. Rep..

[6] Schreyers L., Hauk R., Wallerstein N., Teuling A.J., Uijlenhoet R., van der Ploeg M., van Emmerik T. (2025). Flood type drives river-scale plastic deposition. EarthArXiv.

[7] Schwarz A.E., Ligthart T.N., Boukris E., Van Harmelen T. (2019). Sources, transport, and accumulation of different types of plastic litter in aquatic environments: a review study. Mar. Pollut. Bull..

[8] Hurley R., Braaten H.F.V., Nizzetto L., Steindal E.H., Lin Y., Clayer F., van Emmerik T., Buenaventura N.T., Eidsvoll D.P., Økelsrud A. (2023). Measuring riverine macroplastic: Methods, harmonisation, and quality control. Water Res..

[9] Tasseron P.F., van Emmerik T.H.M., Vriend P., Hauk R., Alberti F., Mellink Y., van der Ploeg M. (2024). Defining plastic pollution hotspots. Sci. Total Environ..

[10] Martínez-Vicente V., Clark J.R., Corradi P., Aliani S., Arias M., Bochow M., Bonnery G., Cole M., Cózar A., Donnelly R. (2019). Measuring marine plastic debris from space: Initial assessment of observation requirements. Remote Sens..

[11] Tasseron P., van Emmerik T., Peller J., Schreyers L., Biermann L. (2021). Advancing floating macroplastic detection from space using experimental hyperspectral imagery. Remote Sens..

[12] Garaba S.P., Dierssen H.M. (2020). Hyperspectral ultraviolet to shortwave infrared characteristics of marine-harvested, washed-ashore and virgin plastics. Earth Syst. Sci. Data.

[13] Knaeps E., Sterckx S., Strackx G., Mijnendonckx J., Moshtaghi M., Garaba S.P., Meire D. (2021). Hyperspectral-reflectance dataset of dry, wet and submerged marine litter. Earth Syst. Sci. Data.

[14] Moshtaghi M., Knaeps E., Sterckx S., Garaba S., Meire D. (2021). Spectral reflectance of marine macroplastics in the vnir and swir measured in a controlled environment. Sci. Rep..

[15] Maathuis M. (2025).

[16] Pérez-García Á., van Emmerik T.H.M., Mata A., Tasseron P.F., López J.F. (2024). Efficient plastic detection in coastal areas with selected spectral bands. Mar. Pollut. Bull..

[17] Tasseron P.F., Schreyers L., Peller J., Biermann L., van Emmerik T. (2022). Toward robust river plastic detection: Combining lab and field-based hyperspectral imagery. Earth Space Sci..

[18] Themistocleous K., Papoutsa C., Michaelides S., Hadjimitsis D. (2020). Investigating detection of floating plastic litter from space using sentinel-2 imagery. Remote Sens..

[19] Garaba S.P., Park Y.J. (2024). Riverine litter monitoring from multispectral fine pixel satellite images. Environ. Adv..

[20] Schreyers L., van Emmerik T., Biermann L., van der Ploeg M. (2022). IGARSS 2022 - 2022 IEEE International Geoscience and Remote Sensing Symposium.

[21] Costa A., Sans E., Pereira-Sánchez I., Duran J., Navarro J. (2024). 2024 International Conference on Machine Intelligence for GeoAnalytics and Remote Sensing (MIGARS).

[22] Nivedita V., Begum S.S., Aldehim G., Alashjaee A.M., Arasi M.A., Sikkandar M.Y., Jayasankar T., Vivek S. (2024). Plastic debris detection along coastal waters using sentinel-2 satellite data and machine learning techniques. Mar. Pollut. Bull..

[23] Sannigrahi S., Basu B., Basu A.S., Pilla F. (2022). Development of automated marine floating plastic detection system using sentinel-2 imagery and machine learning models. Mar. Pollut. Bull..

[24] Cerra D., Auer S., Baissero A., Bachofer F. (2024). Detection and monitoring of floating plastic debris on inland waters from sentinel-2 time series. IEEE J. Sel. Top. Appl. Earth Obs. Rem. Sens..

[25] Mohsen A., Kiss T., Kovács F. (2023). Machine learning-based detection and mapping of riverine litter utilizing sentinel-2 imagery. Environ. Sci. Pollut. Res..

[26] Sakti A.D., Sembiring E., Rohayani P., Fauzan K.N., Anggraini T.S., Santoso C., Patricia V.A., Ihsan K.T.N., Ramadan A.H., Arjasakusuma S., Candra D.S. (2023). Identification of illegally dumped plastic waste in a highly polluted river in indonesia using sentinel-2 satellite imagery. Sci. Rep..

[27] Magyar D., Cserép M., Vincellér Z., Molnár A.D. (2023). Waste detection and change analysis based on multispectral satellite imagery. arXiv preprint.

[28] Molnár A.D., Málnás K., Bőhm S., Gyalai-Korpos M., Cserép M., Kiss T. (2024). Comparative analysis of riverine plastic pollution combining citizen science, remote sensing and water quality monitoring techniques. Sustainability.

[29] Gorelick N., Hancher M., Dixon M., Ilyushchenko S., Thau D., Moore R. (2017). Google earth engine: Planetary-scale geospatial analysis for everyone. Remote sensing of Environment.

[30] Haut J.M., Moreno-Alvarez S., Pastor-Vargas R., Pérez-García Á., Paoletti M.E. (2024). Cloud-based analysis of large-scale hyperspectral imagery for oil spill detection. IEEE J. Sel. Top. Appl. Earth Obs. Remote Sens..

[31] Li J., Wang H., Wang J., Zhang J., Lan Y., Deng Y. (2023). Combining multi-source data and feature optimization for plastic-covered greenhouse extraction and mapping using the google earth engine: a case in central yunnan province, china. Remote Sens..

[32] Pérez-Cutillas P., Pérez-Navarro A., Conesa-García C., Zema D.A., Amado-Álvarez J.P. (2023). What is going on within google earth engine? a systematic review and meta-analysis. Remote Sens. Appl.: Society and environment.

[33] Mohan N., Rao N.S., Ghosh S., Meenakshi (2025). Detection of marine plastics in the north indian ocean with optical and thermal information using google earth engine. Thalassas.

[34] Marye A.T., Caramiello C., De Nardi D., Miglino D., Proietti G., Saddi K.C., Biscarini C., Manfreda S., Poggi M., Tauro F. (2025). Remote sensing for monitoring macroplastics in rivers: A review. WIREs Water.

[35] Acharki S., Veettil B.K., Vizzari M. (2024). Plastic-covered greenhouses mapping in morocco with google earth engine: Comparing sentinel-2 and landsat-8 data using pixel-and object-based methods. Remote Sens. Appl.: Society and Environment.

[36] Rußwurm M., Venkatesa S.J., Tuia D. (2023). Large-scale detection of marine debris in coastal areas with sentinel-2. iScience.

[37] Hoellein T.J., Schwenk B.A., Kazmierczak E.M., Petersen F. (2024). Plastic litter is a part of the carbon cycle in an urban river: Microplastic and macroplastic accumulate with organic matter in floating debris rafts. Water Environ. Res..

[38] Schreyers L.J., van Emmerik T.H.M., Bui T.K.L., Biermann L., Uijlenhoet R., Nguyen H.Q., Wallerstein N., van der Ploeg M. (2024). Water hyacinths retain river plastics. Environ. Pollut..

[39] Erni-Cassola G., Zadjelovic V., Gibson M.I., Christie-Oleza J.A. (2019). Distribution of plastic polymer types in the marine environment; a meta-analysis. J. Hazard. Mater..

[40] Meijer L.J.J., Van Emmerik T., Van Der Ent R., Schmidt C., Lebreton L. (2021). More than 1000 rivers account for 80% of global riverine plastic emissions into the ocean. Sci. Adv..

[41] Pinto R.B., Bogerd L., van der Ploeg M., Duah K., Uijlenhoet R., van Emmerik T.H.M. (2024). Catchment scale assessment of macroplastic pollution in the odaw river, ghana. Mar. Pollut. Bull..

[42] Rinasti A.N., Ibrahim I.F., Gunasekara K., Koottatep T., Winijkul E. (2022). Fate identification and management strategies of non-recyclable plastic waste through the integration of material flow analysis and leakage hotspot modeling. Sci. Rep..

[43] Pinto R.B., van Emmerik T.H.M., Duah K., van der Ploeg M., Uijlenhoet R. (2024). Mismanaged plastic waste as a predictor for river plastic pollution. Sci. Total Environ..

[44] Van Dalen J., Asano Y.M., Rußwurm M. (2025). Samselect: A spectral index search for marine debris visualization using segment anything. IEEE Geoscience and Remote Sensing Letters.

[45] Papageorgiou D., Topouzelis K., Suaria G., Aliani S., Corradi P. (2022). Sentinel-2 detection of floating marine litter targets with partial spectral unmixing and spectral comparison with other floating materials (plastic litter project 2021). Remote Sens..

[46] Dittmann S., Kiessling T., Mederake L., Hinzmann M., Knoblauch D., Böhm-Beck M., Knickmeier K., Thiel M. (2023). Sharing communication insights of the citizen science program plastic pirates—best practices from 7 years of engaging schoolchildren and teachers in plastic pollution research. Front. Environ. Sci..

[47] Van Emmerik T., Roebroek C., De Winter W., Vriend P., Boonstra M., Hougee M. (2020). Riverbank macrolitter in the dutch rhine–meuse delta. Environ. Res. Lett..

[48] Planet (2025). Planet Documentation.

[49] Pahlevan N., Sarkar S., Franz B.A., Balasubramanian S.V., He J. (2017). Sentinel-2 multispectral instrument (msi) data processing for aquatic science applications: Demonstrations and validations. Remote Sensing of Environment.

[50] Breiman L. (2001). Random forests. Mach. Learn..

[51] Ho T.K. (1995). Random decision forests. Proceedings of 3rd International Conference on Document Analysis and Recognition.

[52] Carlson T.N., Ripley D.A. (1997). On the relation between ndvi, fractional vegetation cover, and leaf area index. Remote Sensing of Environment.

[53] Biermann L., Clewley D., Martinez-Vicente V., Topouzelis K. (2020). Finding plastic patches in coastal waters using optical satellite data. Sci. Rep..

