# Supplementary material for: River plastic hotspot detection from space
Source: iScience. 2025 Dec 29;29(2):114570. doi: 10.1016/j.isci.2025.114570 (PMC12828594; doi:10.1016/j.isci.2025.114570)
Supplement: Document S1. Table S1 [file mmc1.pdf]

iScience, Volume *29*

Supplemental information

River plastic hotspot detection from space

Ámbar Pérez-García, Graciela Amanda, José F. López, Marc Rußwurm, and Tim H.M. van Emmerik

Table 1: Sentinel-2 dataset with the number of pixels identified per class.

#	Date	ID	Pixels		
			**Water**	**Vegetation**	**Plastic**
		**Indonesia**			
1	02/08/2021	20210802T025551_20210802T030951_T48MYT	137	10	46
2	17/07/2024	20240717T025551_20240717T031431_T48MYT	95	12	55
3	18/06/2023	20230618T025529_20230618T030925_T48MYT	254	27	279
4	08/07/2022	20220708T025541_20220708T031928_T48MYT	137	7	87
5	12/08/2021	20210812T025551_20210812T030951_T48MYT	38	17	155
6	02/08/2022	20220802T025529_20220802T031001_T48MYT	41	6	37
7	24/05/2020	20200524T025549_20200524T031633_T48MYT	57	3	321
8	13/06/2023	20230613T025531_20230613T030737_T48MYT	281	12	47
9	13/06/2020	20200613T025549_20200613T031826_T48MYT	233	3	44
10	17/06/2024	20240617T025531_20240617T031846_T48MYT	524	149	376
		**Guatemala**			
1	04/04/2020	20200404T161829_20200404T162814_T15PYS	31	7	89
2	03/05/2024	20240503T161829_20240503T163152_T15PYS	23	16	12
3	19/04/2022	20220419T161831_20220419T163150_T15PYS	28	23	70
4	30/11/2022	20221130T162629_20221130T163644_T15PYS	73	42	36
5	19/05/2023	20230519T161829_20230519T163309_T15PYS	38	25	86
6	25/03/2021	20210325T161921_20210325T163554_T15PYS	58	17	39
7	10/12/2020	20201210T162659_20201210T163918_T15PYS	56	47	65
8	09/11/2024	20241109T162409_20241109T163627_T15PYS	45	35	81
9	03/02/2021	20210203T162501_20210203T163338_T15PYS	78	49	78
10	29/01/2023	20230129T162529_20230129T162713_T15PYS	61	28	37
		**Ghana**			
1	29/03/2020	20200329T101021_20200329T101946_T30NYM	18	0	33
2	02/03/2023	20230302T101839_20230302T103621_T30NZM	27	0	49
3	11/01/2021	20210111T102309_20210111T103617_T30NZM	14	0	29
4	14/03/2022	20220314T101019_20220314T102151_T30NYM	51	0	38
5	11/01/2024	20240111T102401_20240111T102734_T30NZM	57	0	136
6	19/12/2023	20231219T101431_20231219T102022_T30NZM	34	0	17
7	29/11/2020	20201129T101359_20201129T102206_T30NZM	19	0	10
8	18/03/2024	20240318T100731_20240318T102712_T30NYM	40	0	55
9	19/11/2022	20221119T101229_20221119T102030_T30NZM	54	0	29
10	10/02/2021	20210210T102049_20210210T103356_T30NZM	21	0	30
